# Quantitative peel test for thin films/layers based on a coupled parametric and statistical study

**DOI:** 10.1038/s41598-019-55355-9

**Published:** 2019-12-24

**Authors:** Maysam Rezaee, Li-Chih Tsai, Muhammad Istiaque Haider, Armin Yazdi, Ehsan Sanatizadeh, Nathan P. Salowitz

**Affiliations:** 1 Department of Mechanical Engineering, University of Wisconsin-Milwaukee, Milwaukee, USA; 2 Department of Biomedical and Health Informatics, University of Wisconsin-Milwaukee, Milwaukee, USA; 3 Department of Civil & Environmental Engineering, University of Wisconsin-Milwaukee, Milwaukee, USA

**Keywords:** Mechanical engineering, Statistics

## Abstract

The adhesion strength of thin films is critical to the durability of micro and nanofabricated devices. However, current testing methods are imprecise and do not produce quantitative results necessary for design specifications. The most common testing methods involve the manual application and removal of unspecified tape. This overcome many of the challenges of connecting to thin films to test their adhesion properties but different tapes, variation in manual application, and poorly controlled removal of tape can result in wide variation in resultant forces. Furthermore, the most common tests result in a qualitative ranking of film survival, not a measurement with scientific units. This paper presents a study into application and peeling parameters that can cause variation in the peeling force generated by tapes. The results of this study were then used to design a test methodology that would control the key parameters and produced repeatable quantitative measurements. Testing using the resulting method showed significant improvement over more standard methods, producing measured results with reduced variation. The new method was tested on peeling a layer of paint from a PTFE backing and was found to be sensitive enough to register variation in force due to differing peeling mechanisms within a single test.

## Introduction

Small-scale devices are increasingly prevalent, enabling new technologies and changing our world. Micro and nanofabrication techniques have been employed to make micro-processors, sensors, actuators, and other devices that have been integrated into telephones, computers, cars, medical instruments, and other apparatus. Common micro and nano-fabrication techniques employ depositing and/or removing multiple thin layers of material to simultaneously create large numbers of devices. These devices are increasingly becoming portable, mobile, and flexible requiring them to survive physical loads and abuse that were not previously a major concern. Modern small electronics need to survive being shipped, dropped, sat upon and used in other ways.

Current methods to test mechanical survival of microfabricated devices are crude and imprecise. Protective packaging is commonly created in conjunction with micro devices to protect from contact and other potential damage or bonding layers are used to *improve* adhesion between layers (by an unspecified amount)^1^.

One of the most common ways to test bonding between microfabricated layers is known as ‘the tape test’. This test is an adaptation of ASTM D3359 “Standard Test Methods for Measuring Adhesion by Tape Test”^2^. ASTM D3359 involves cutting a pattern of lines in the layer to be tested which is often unnecessary when testing microfabricated devices. Tape is manually applied to the test layer and then rapidly removed. ASTM D3359 has the advantage of employing readily available supplies without significant equipment. However, this test has many limitations; (1) while ASTM D3359 recommends a specific tape, the recommended tape is no longer available and alternates are not specified. (2) Manual application of the tape is poorly controlled with no specified application pressure or duration. A wait time between application and peeling is specified but has significant variation. (3) The peel rate is not specified or controlled, (4) The test does not produce a quantitative measurement in scientific units, but rather a numerical ranking based on visual assessment. Experimental results presented within this paper indicate that parameters including the magnitude and duration of tape application pressure, time waited between tape application and testing, and peel rate all can lead to significant variations in the tape adhesion strength. Combined, these issues result in low precision with ASTM D3359 reporting variation in results within-laboratories and between-laboratories are 37% and 70% respectively.

Other methods have been used to measure the adhesion strength of thin films, nano-scale layers, or particles dependent on their intended applications. Pull-off tests are one the most popular adhesion test for paint and metal coatings on quasi-rigid substrates like steel, concrete, or wood. This method is specified in ASTM D-4541 and D-7234, DIN EN ISO 4624^3–6^ and is usually performed on macro scale applications like evaluation of bonding strength of coatings or surface treatments on structural concrete or steel members. The shear lap test is another mechanical test that quantifies the shear strength of adhesion through imposing shear force on the adhesion interface. This method is specified by ASTM D-1002, D-3163-01, and D-5868-01^7–9^. In this test, two quasi-rigid substrates are bonded together and then pulled in opposite directions. The stiffness of the substrates is extremely important to generating nearly pure shear during the test^10^ which makes this method unsuitable for flexible substrates. The shear lap test is applicable for macro/micro applications and is frequently used to assess structural bonding as well. Three and four-point bending tests are normally used as a method for assessment of bonding of layers of composite members. Four-point bending test is very similar to three-point bending test but adding an extra contact point results in lower bearing stress for a given load. This method is also specified by ASTM D-1624-05 and D-7249^11,12^. These tests place adhesive layers in transverse shear, coupled to bending, to test the bond strength. The scribe test, also known as the scratch test^13^, is performed through applying a normal force on a stylus tip to scratch a coated surface in parallel line or rectangular grid patterns with a minimum spacing of 0.4 mm. If any coating between the lines breaks away from the substrate, the adhesion is considered inadequate^14^. The test can be implemented on macro/micro/nano-devices^15^ and used to evaluate the bonding performance of brittle thin films^16^. This method could be affected by many factors including loading rate, scratching speed, tip shape, the hardness, roughness, thickness of coatings. The blister test is used to measure the adhesion of thin films by imposing of outward pressure on the thin film. Employing fracture mechanics and membrane theory, this method estimates the energy release rate^17–19^. The blister method requires a complex test setup and can be challenging when applied to very thin ductile films (<2 μm)^19^. The micro/nano-indentation tests are normally used for the measurement of hardness and elastic moduli of thin films^19,20^ by using Vickers and Berkovish shaped tips and determining the strength of interfacial fracture from delamination. According to ISO 14577, the test is considered micro indentation when the force is less than 2 N and indentation depth more than 0.2 μm and the test is considered nano indentation when indentation depth is less than 0.2 μm. The indentation is measurement of peak force over contact area to obtain the hardness. The drop test^21^ is an experimental method that is easily performed based on the momentum of particles adhered to a surface and then dropped from a specific height and abruptly stopping upon landing on a test fixture. The adhesive force and energy between the particles and the substrate surface can be calculated through Johnson-Kendall-Roberts (JKR) theory of adhesive contact using a balance between the stored elastic energy and the loss of surface energy. Since it is based on microscopic observations of the particles, the method is considered robust.

There are a range of devices available to measure the adhesion strength of macro scale thin films. These devices have the disadvantages of being expensive and only being able to measure films’ bonding over large areas (on the order of square cm minimum). Unfortunately, aside from the devices designed to measure the adhesion strength of large-scale films, methods do not provide direct quantitative measurements of adhesion strength. Additionally, there is an increasing interest in devices created on flexible substrates^22–24^, further complicating and adding error to test methods. As a result, quantitative design specifications cannot be created or met without significant testing and over designing.

Small-scale thin films are inherently hard to mechanically test because of their size. There is little structure to attach to and the small magnitudes of their properties makes measurement challenging. The objective of this work was to create a simple, accurate, and easily performed technique to quantitatively measure the bonding strength between thin-films/layers and underlying structure employing commonly available supplies and tools and producing repeatable results.

## Methodology

**Figure 1:**
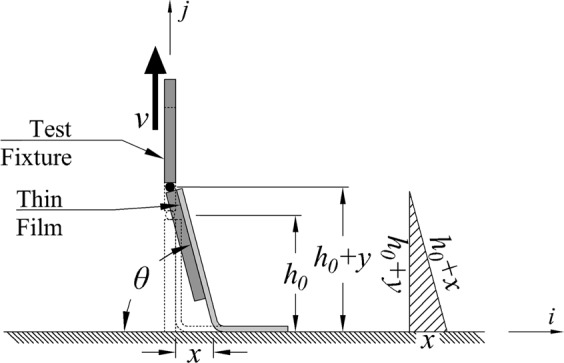
Schematic view of straight pull tests that starts with initial peel arm of *h*_*0*_ and peel angle of 90°. The peel rate ($\dot{{x}}$) and peel angle (*θ*) change nonlinearly during test that the hatched triangle demonstrates the relationship between pull rate (v), peel rate ($\dot{{x}}$), peel angle (*θ*), and vertical movement of tape extremity (*y*).

Experiments and analysis were employed to identify parameters that cause variation in dynamic peel strength of films and tapes. Based on the results, a testing method was created to control the key parameter, minimize variation, and produce consistent quantitative results. The method created sought to employ common and readily available laboratory equipment in order to maintain ease of use and tape continued to be used as the medium to adhere to the films for testing, however, quantitative measurement of the peel force negates issues with using different types of tape. In the new test method different types of tape can be used advantageously to adjust the range of peel strength tested. Literature, analysis, and initial testing identified the peel rate and angle as significant sources of variation in the force required to peel tape from a substrate. Peel angle is defined as the angle measured between the free tape being pulled and the bare surface, opposite where the tape is adhered to the sample, represented by (*θ*) in Fig. 1. It has been reported that the relation between the peel force and the peel rate follows a power law equation at a constant peel angle of 90°^25–28^. Even with constant peel rate, variation in the peel angle causes dramatic variation of peel force such that the peel force significantly changes from peel angle of zero to 90 degree^25,27–31^ and beyond the 90 degree of peel angle, a minor variation of peel force is expected.

An experimental setup was created to control the rate and angle of peeling tape from a sample while precisely measuring the force generated by employing automation, kinematics, and sensors. The experimental setup was then used to perform testing and a factorial experimental analysis to identify other parameters that affect the adhesion strength of the tape. Based on the results of the factorial analysis, methods were created to standardize and control parameters that affected tape adhesion strength in order to produce repeatable quantitative measurements. Once repeatable quantitative measurements were achieved, the techniques were tested on thin films to measure the peel force, which represented one of: (1) bonding strength between thin film and substrate (adhesive or adherent failure), (2) cohesive failure that shows the interlayer debonding of the thin layer, (3) partial bonding strength that would be combination of adhesive, bonding, and cohesive failures.

For the purposes of this study, the peel rate is defined as the velocity of the peel front between the tape or film and the substrate. The peel angle is defined as the angle formed between the surfaces of tape (or thin film) being pulled away and the substrate. The pull rate is the velocity of the extremity of the tape opposite the point of separation from the substrate, in the frame of reference of the substrate but not necessarily aligned with the tape or substrate.

### Experimental setup

**Figure 2:**
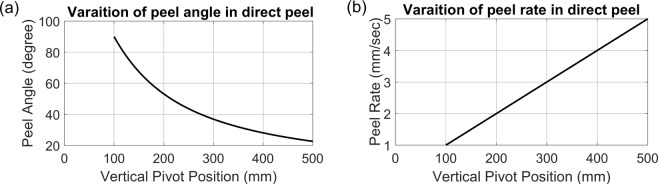
Variation of peel angle and peel rate for straight peel test. (**a**) The peel angle (*θ*)) changes with a nonlinear trend versus vertical movement of extremity of tape. (**b**) Peel rate has linear change while the extremity goes away in vertical direction. This plots are drawn for a nominal *h*_*0*_ = 100 mm and a constant pull rate *v* = 1 *mm*/*sec*.

An Instron 3369 dual column tabletop testing system was employed to produce consistent and controllable pull rates. Basic geometries (Fig. 1) were found to have non-constant, non-linear relations between pull rate (*v*) and both peel front velocity ($\dot{x}$) and peel angle (*θ*) as shown in Fig. 2(a,b). The following presents theory highlighting the nonlinearity of these properties in basic geometries followed by a generalizable theory that enabled the design of a simple, static jig that would linearly scale pull rate to peel rate and maintain a constant peel angle. The tensile test system was equipped with an Omega LCL-010 full bridge load cell to measure the loads. This was intended to provide quantitative measurement of the force required to peel the tape off of a specimen, independent of the type of tape or ambient conditions.

### Basic straight pull: non-linear rate and variable angle

As a reference, the properties and relations are presented for peeling tape off of a stationary horizontal surface (aligned with the <*i*> axis), with an initial peel angle of 90° and vertical <*j*> pull direction, perpendicular to the surface and initially aligned with the tape, resulting in horizontal <*i*> movement of the separation point. The horizontal <*i*> movement of the peel front by distance of $x=y+({y}^{2}/2{h}_{0})$ causes the relative peel angle of $\theta ={\tan }^{-1}(({h}_{0}+y)/x)$ and peel rate of $\dot{x}=v(1+y/{h}_{0})$ to vary nonlinearly with time as well as reorienting the peel force vector. These quantities can be geometrically related as shown in Fig. 1. In the equations for peel rate and angle, *h*_0_ is the initial free length of tape (or initial vertical position of pivot) and *y* is the vertical movement of that point.

The equations and examples shown in Fig. 2(a,b) clearly show that both the peel rate and the angle change significantly at a constant pull rate in this configuration.

Employing larger *h*_0_ would reduce the nonlinearity. As *h*_0_ approached infinity, *x* and *θ* will approach *y* and 90° respectively, and $\dot{x}$ would approach *v*. It was thought that this approach could be implemented within measurement tolerance. Testing revealed that this approach required large test setups (*h*_0_ > 500 mm) that introduced dynamic problems and other issues. Additionally, it has been noted that even small variations in peel angle have a dramatic effect on peel force^27,28,32^. Therefore, other approaches were explored.

### Angle control

**Figure 3:**
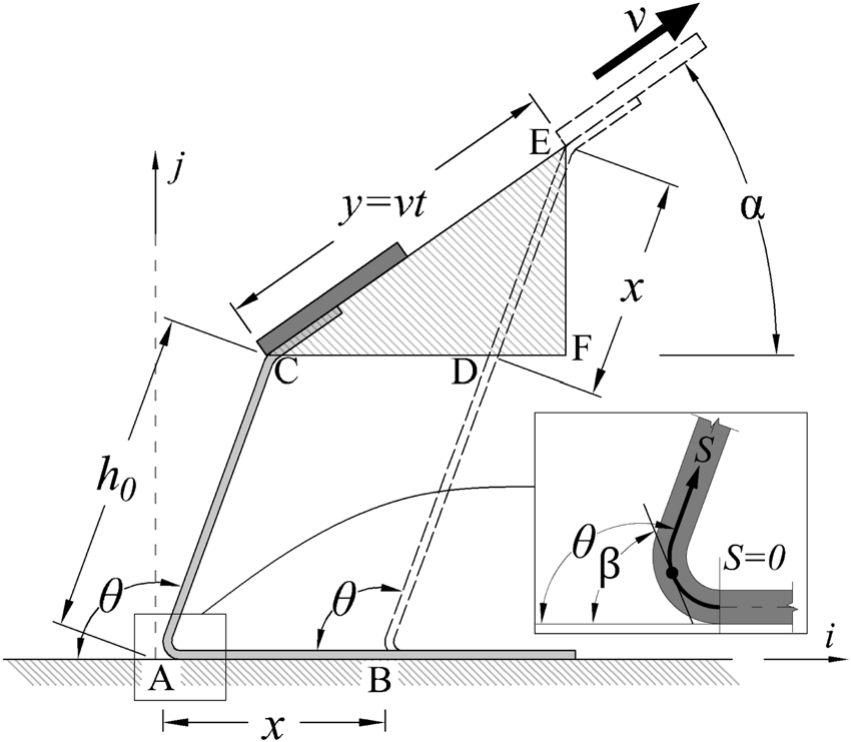
Geometric view of controlled peel test in which the constant peel angle of *θ* (and sequentially the constant peel rate of *v*) requires the pull force to be applied in angle of *α*.

**Figure 4:**
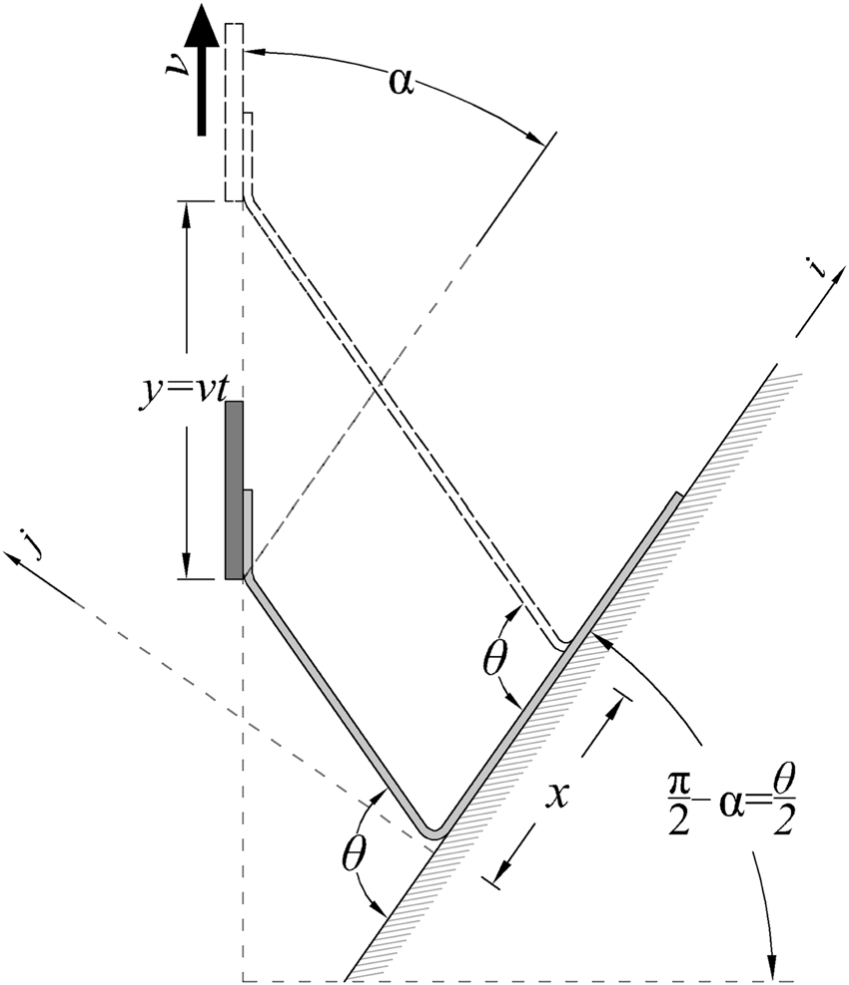
The substrate must be rotated by the angle of *θ*/2 since the pull force is applied just in vertical direction.

A geometric analysis was performed to identify pull angle (*α*) that would produce constant peel angle (*θ*) and peel rate ($\dot{x}$) for an ideally inextensible thin film (or peel arm). In case of small pull force and moderate peel angle, the assumption inextensibility of peel arm is reasonable^25,33–37^. Considering the peel front movement in Fig. 3, the relationship of peel front movement and extremity movement can be obtained by $x=y[\,\cos \,\alpha /(1-\,\cos \,\theta )]$. Triangle DEF, $x=y(\sin \,\alpha /\,\sin \,\theta )$ provides another relation between *x* and *y*. Combining equations for *x* and treating y as a constant as desired, the desired pull angle can be calculated as $\alpha =(\pi -\theta )/2$ which creates constant peel rate and peel angle. For testing purposes, a jig was constructed that would rotate the frame of reference to align the pull direction vertically, for the tensile tester, and provide a desired slope for the substrate. As shown in Fig. 4, the jig angle equals to ($\pi /2-\alpha$) which is equivalent to *θ*/2. Based on this, a jig and test setup were constructed for further testing and analysis, with a slope of 45° to maintain a 90° peel angle.

The 90° peel tests studied in this paper relates the measured pull force (*F*_*pull*_) per width (*W*) of peel arm to a corresponding energy release rate of ${F}_{pull}(1-\,\cos \,\theta )/W$, as originally introduces by Rivlin^38^. Also, It has been reported that the relation between the peel force and the peel rate follows a power law equation at a constant peel angle of 90°^25–28^. Based upon a discretized model suggested by Xia *et al*.^34^, the peel force is significantly affected by peel angle as following:

1 $$\varepsilon =\mathop{\sum }\limits_{i=1}^{N}{\int }_{{s}_{i-1}}^{{s}_{i}}\frac{1}{2}{D}_{i}{(\beta ^{\prime} (s))}^{2}ds-{\int }_{0}^{{s}_{N}}{F}_{pull}(\cos \,(\beta -\theta )-1)\,ds-{\int }_{0}^{l}G\,ds$$

Three terms in above equation show works done by bending of peel arm, pull force, and adhesion energy respectively where *ε* is the potential energy, *D*(*s*) is the distribution of bending rigidity, and G is the constant adhesion energy. In addition to the peel angle (*θ*), the lengthwise angle of peel arm (*β*) is another significant factor on potential energy^36^ which is corresponding to the peel front angle at *s* = 0 as represented in Fig. 3. Therefore, a variation in the peel angle causes dramatic variation of peel force such that the peel force significantly changes from peel angle of zero to 90 degree^25,27–31^ and beyond the 90 degree of peel angle, a minor variation of peel force is expected. On the other hand, for peel angle of smaller than 90 degrees, the contribution of the fracture mode II is notably high that leads to increasing of peel force.

## Results

Tests were performed with the test setup that would enable simple control of peel rate and angle while measuring the force applied to peel the tape. Test results were inspected individually to determine trends and approaches to produce consistent results. An additional series of tests with a factorial analysis was also performed to identify any combinatorial effects. All results presented here, unless otherwise specified, were produced with 3 M Scotch® Magic™ tape 810 (with the width and total thickness of 19 mm and 0.060 mm respectively) on PolyTetraFluoroEthylene (PTFE) and PolyEther Ether Ketone (PEEK) substrates. Substrates were chosen because of their different surface bonding characteristics and prevalence. Across all tests, global environmental conditions like temperature or humidity were ambient and considered constant because the tests were performed consecutively.

### Peel rate

**Figure 5:**
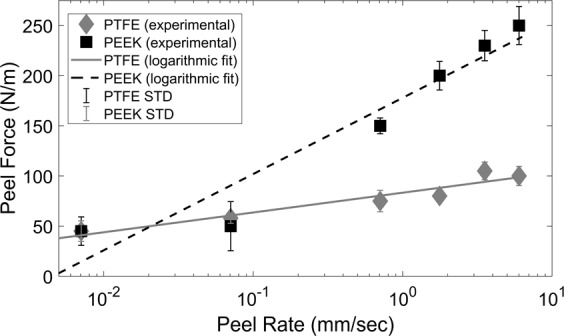
A logarithmic trend of peel force variation versus peel rate for both PTFE and PEEK demonstrating that the peel force is a highly rate-dependent parameter.

It is experimentally demonstrated that higher peel rates cause higher peel force^25,39–41^. The primary reason is changing the effective length of cohesive zone in the adhesive layer which is changing dramatically by the peel rate. Then, the longer cohesive zone is corresponding to higher adhesion energy^42^ and sequentially higher peel force. As mentioned in the Methodology section, of the factors tested, the peel rate had the largest impact on the peel force. Multiple tests were performed with controlled, constant peel rates ranging from 0.01 to 10 mm/sec. The range was limited at the low end by the sensitivity of the load cell, and at the high end by the capabilities of the Instron. Inspection of the results presented in Fig. 5 found that the relation between the average peel force and peel rate followed a logarithmic trend with coefficients of determination (R^2^) of 91.2% and 92.0% respectively. This result confirms that peel rate must be precisely controlled in order to produce repeatable results. Additionally, this highlights the potential to intentionally use different peel rates to produce different peel forces. The trends of peel force variation versus peel rate have validated previously reported trends as explained in literature^25–28^.

### Magnitude of application pressure

Although, a few papers mentioned the application pressure as a significant factor on the peel force^40,43,44^, the effects of application pressure on the peel force has not been studied individually.

Imposing the pressure on tape causes more uniformity of adhesive layer through removing small air bubbles and decreasing the adhesive heterogeneity. Because of local variation of peel front angle (*β*), the most highlighted effect of adhesive heterogeneity is dominant jumps and drops of peel forces^45–47^ and variations in average peel force which in not desirable in peel test.

**Figure 6:**
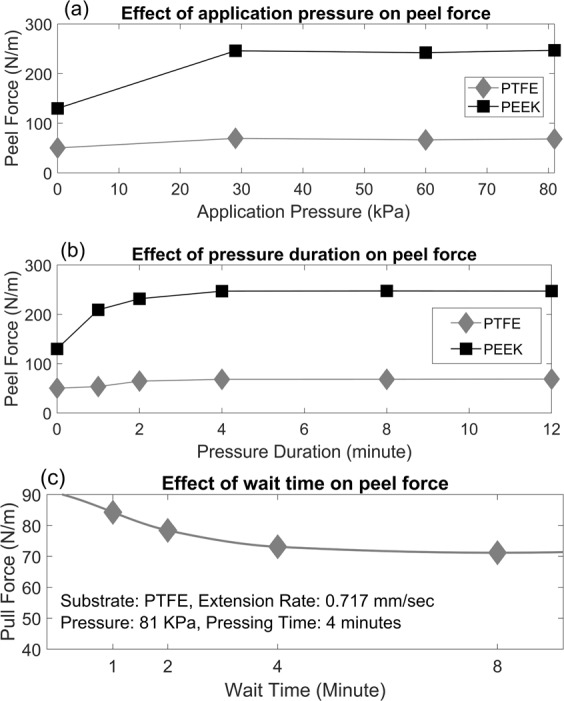
Significant factors on peel force; (**a**) application pressure effect on the peel force for PTFE and PEEK; in these experiments the peel tests are done after application of pressures for 8 minutes including 4 minutes of pressure duration and 4 minutes of wait time, (**b**) application duration effect on the peel force for PTFE and PEEK is evaluated with pressure of 81kPa in addition to 4 minute of wait time, and (**c**) wait time effect on peel force for PTEF is assessed considering pressure of 81 kPa during 4 minutes.

Tests on the effects of different magnitudes of application pressures on the peel force of tapes were performed using press pads to uniformly distribute applied forces. Magnitudes of: 0, 29, 60, and 81 kPa were applied with the other typical parameters producing the peel force results shown in Fig. 6(a). The results show that the magnitude of pressure when adhering tape to a substrate can have a large effect on the peel force up to a point, above which the pressure does not alter the peel force. No effects on the peel force were found with application pressures greater than 30 kPa, therefore, 30 kPa is recommended as a minimum application pressure. In further testing presented herein 81 kPa was selected as an application pressure to help remove air bubbles and enhance interfacial adhesion for consistent results.

### Duration of application pressure

Tests on the effects of the duration of application pressures on the peel force of tapes for durations of 1, 2, 4, 8, and 12 minutes producing the results shown in Fig. 6(b). The results show that the variation in peel force is considerable for pressure durations up to 4 minutes, beyond which the peel force showed negligible variation. Based on this result, a minimum pressure application time of 4 minutes was selected to minimize variation.

### Waiting time

The wait time between the removal of the application pressure and initiation of peeling was also varied to determine its effect on peel force. It required a minimum of one minute to remove the pressure and then mount the specimens into the testing machine so that was the minimum wait time tested. Actually, during the wait time, the adhesive layer comes back to the normal state with more uniformity and more guaranteed reputability. As shown in Fig. 6(c), the wait time also has an effect on the peel force, though it is smaller than the effects of the magnitude of application pressure and duration. Like magnitude and duration of the application pressure, the wait time appeared to approach a constant value with little variation beyond 4 minutes. This suggests that the tape relaxed after removing the application pressure and waiting a minimum of 4 minutes would reduce potential variation in peel strength due to the wait time. A few studies mentioned the wait time in different ranges like a few minutes^26^, 20 minutes^25^, and a day^40^.

### Factorial analysis

A series of experiments and multi-level factorial analysis were performed to rank the influence of the parameters tested and identify any combinatorial effects on the adhesion/peel strength of tape. The 45° test jig was used to eliminate the effects of peel angle and peel rate by keeping them constant across experiments. These experiments addressed the magnitude of tape application load, duration of application load, and peel rate at three levels each as is shown in Table 1. The experiments used 3 M scotch® Magic™ 810 tape on PTFE and PEEK substrates. The effect of wait time was significantly smaller than the other factors, providing a maximum variation of 15 N/m compared to hundreds of N/m for the other factors. Therefore, wait time was kept at a constant 4 minutes and not varied in the factorial analysis.

**Table 1:** The three levels factorial analysis parameters.

Parameter	1^st^ Level	2^nd^ Level	3^rd^ Level
Velocity (mm/sec)	0.141	0.707	3.535
Pressure (kPa)	29	60	81
Duration (minutes)	1	2	4

**Figure 7:**
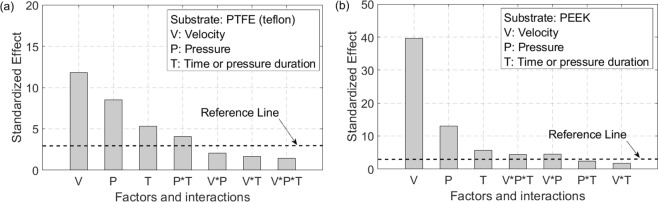
Normal effects plots showing all three factors of peel rate, application pressure, and pressure duration have a significant effect on peel force whereas interactions are not significant. (**a**) Plot for PTFE substrate and (**b**) plot for PEEK substrate.

The peel rate or velocity was tested at magnitudes of 0.141, 0.707, and 3.535 mm/sec, controlled by the Instron MTS machine. A rubber press pad was employed to distribute the applied pressure uniformly across the tape and substrate with applied pressures of 29, 60, and 81 kPa. These pressures were applied for durations of 1, 2, and 4 minutes. Based on the analysis of variances done for the peel force, the peel rate has the strongest effect on the peel force, followed by the magnitude of the application pressure and the duration of the application pressure. These are confirmed by the Pareto charts based on a 3 level factorial analysis of the extreme values shown in Fig. 7(a,b) for both substrates. The reference lines in Fig. 7 identify the significance level of each factor in factorial analysis such that factors with lower effect than reference line are not statistically significant. Combinatorial interactions of the 3 parameters studied were found to be smaller than any of the individual effects according to the factorial analysis

### Consistency and evaluation of proposed setup

As shown in the prior sections, the magnitude of the peel force was affected by the duration of applied pressure, magnitude of applied pressure, and wait time between removing the application pressure and testing. Each of these parameters approached critical values beyond which variation was nominal providing minimums to produce consistent results. Based on the results of this testing, a pressure with a minimum magnitude of 30 kPa should be applied for a minimum duration of 4 minutes, a minimum of 4 minutes should elapse between the pressure removal and peel testing to produce consistent forces. Additionally, peel rate and peel angle have drastic effects on the peel force and therefore must be controlled.

**Figure 8:**
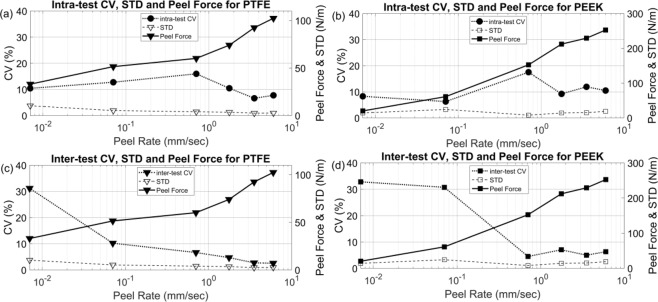
Coefficient of variations are calculated through experiments demonstrating an acceptable consistency of result given form proposed setup for peel test. (**a**,**b**) The intra-test CV trends changed to a decreasing trend after peel rate of 0.707 mm/sec for both PTFE and PEEK demonstrating that the asynchronous failures of filaments of adhesive layer is much lower in for peel rate around 1 mm/sec and beyond; (**c**,**d**) an inverse trend of inter-test CV in comparison to peel force is obtained for both PTFE and PEEK demonstrating higher consistency of proposed test method at higher peel rates; For all plots the standard deviation is roughly similar showing that the significant factors on peel force are well-controlled.

To evaluate the effectiveness of the combined proposed testing method another series of test was performed with different peel rates using the recommended minimum times and pressure of 81 kPa, well above the minimum recommended to help reduce air bubbles and inconsistencies. Two different coefficients of variation (CVs) were calculated for the tests: (1) an intra-test CV (as shown by dotted line in Fig. 8(a,b) was calculated for the force generated in the constant peel region in a single test. The intra-test CV provides a measure of the variation that occurs in peeling a single piece of tape from a single substrate with a single release mechanism. (2) an inter-test CV (as presented by dotted line in Fig. 8(c,d)) was calculated across the mean forces produced by multiple similar tests. The inter-test CV evaluates the variability of the average of the peel force in the region with steady state forces for similar tests. As can be seen in the data in Table 2 and Fig. 8(a–d), higher peel rates had a higher consistency and lower CVs for both analyses with both substrates. Closer examination revealed that the standard deviation of the peel forces remained relatively constant across peel rates while the mean peal force increased with peel rates resulting in a reduction in the CV with increased peel rate. Looking at the cohesive zone in micro scale, at lower peel rates, the filaments of adhesive layer behave more actively which leads to dominant asynchronous failures of filaments along the width of the tape. Therefore, in lower peel rates, these irregular failures of filaments increase the variation of the peel force for each test in the steady state region. The average magnitude of inter-test CV for PTFE and PEEK are about 6 and 8 percent respectively, and are smaller at higher peel rates demonstrating improved consistency compared to the reported magnitude variation of 37% by ASTM D-3359^2^.

**Table 2:** inter-test and intra-test CVs for PTFE and PEEK.

Substrate	CV (%)	Peel Rate (mm/sec)					
		0.007	0.071	0.707	1.768	3.353	6.010
PTFE	intra-test	8.270	6.257	17.530	9.203	11.913	10.480
	inter-test	21.444	10.640	16.966	5.575	8.439	11.125
PEEK	intra-test	10.423	12.737	15.963	10.453	6.610	7.743
	inter-test	32.843	30.745	4.492	7.028	4.966	6.304

### Testing peeling of a thin film

**Figure 9:**
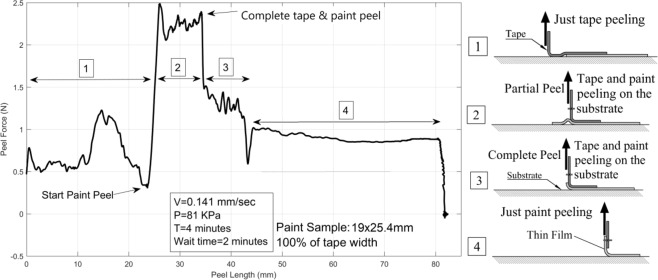
A layer of 1 by 1 in. of Latex paint on PTFE substrate is tested and for phases of peeling were observed; (1) peeling of tape from substrate, (2) partial peeling of tape and paint from substrate which is included with extra bending and elongation of paint layer. These led to variable local peel angle that makes this case sophisticated, (3) peeling of tape and paint from substrate and (4) peeling of paint layer from substrate which is much more consistent that other modes because of lack of adhesive layer.

The peel testing method presented herein was performed on thin films applied to PTFE substrates to further evaluate their capabilities. In the test Behr latex paint was applied to PTFE to create a thin film that would peel off of the substrate with an adhesive failure. Four hours after deposition, samples measuring 19 × 25.4 mm (3/4 by 1 inch) were tested with a tape application pressure of 80 kPa for a duration of 4 minutes, a 4 minute wait time, and a peel rate of 0.141 mm/sec. Four phases of peeling were observed, corresponding to 4 different peel forces as shown in Fig. 9. In the first phase, tape was directly adhered to, and peeled off of the substrate. In the second phase the tape was peeling off of the film and the film was also peeling off of the substrate forming a little bubble under the film as shown in Fig. 9. The complex adhesion and peeling geometry in phase 2 showed the highest peel force in comparison to other phases due to two contributing factors: (1) the bending of both tape and plastic film and (2) extra bending and extension of the film with a partial connection to the substrate which also created an effective angle change. Phase 3 began when the film detached from the substrate at one end and began a complete peel from the substrate at a single point. Phase 3 had a lower peel force than phase 2 but higher than phase 1. In this phase the plastic film was peeling from substrate but both the tape and film were bending. In phase 4, the plastic film was peeling from the substrate without tape backing it. A big drop of measured force occurred at the start of phase 4 due to the low axial stiffness of the film resulting in a large increase in strain, i.e. the film elongated with minimal peeling. This effect disappeared quickly and the peel rate returned to the initial value regenerating the peel force, which was consistent for the remainder of phase 4. The differences in the phases highlights the sensitivity of the method developed, even detecting the force required to bend the tape. The lack of variation in phase 4 compared to the other phases was attributed to the change in peel mechanism. In phase 1 the tape was peeling directly off of the substrate and a roughly randomized failure of filaments of the adhesive layer at the peel front region caused variations in the peel force^35,36,45,48^. Similarly, phase 2 was an unstable phase with multiple modes of peeling and slightly changing angles due to the bubble under the film. Phase 2 and 3 also had effects of bending a multi-layered beam with viscously bonded layers. Phase 4 consisted exclusively of the dry film bending and peeling off of the substrate without the soft and variable adhesive layer which created a relatively constant force.

## Conclusions

This paper presented a study to produce a simple and broadly applicable test to quantitatively measure peel forces using commonly available laboratory equipment and supplies. Parameters that affect the peel force were identified and evaluated to control their impact on results. Peel rate and angle were identified as having major impacts on the peel force. Therefore, designs and relations to easily control the rate and angle of peeling, and keep them constant, were created. Magnitude and duration of application pressure and wait time before peeling were also tested. Analysis found that the effects of application pressure and its duration and the time from pressure removal to test start, all had significant effects on the adhesion force, but factorial analysis indicated that combinatorial effects were smaller. Further inspection found that all of the parameters approached asymptotes, with longer wait times and larger pressures producing more consistent results and less variation. It is worth noting that some of the experimentally observed trends can explain some of the inconsistency observed in established, standard, tests because the parameters are poorly controlled or the specification spans a range with large variation. Considering the effects of previously mentioned significant parameters on the peel force, threshold values of these parameters were identified to reduce the variability and improve the consistency of measurements.

Experiments were performed with tape applied directly to PTFE and PEEK as well as peeling a latex plastic film off of PTFE. These tests employed a peel angle of 90°, tape application pressure of 81 kPa applied for 4 minutes and a wait time of 4 minutes. These tests produced relatively consistent results with average inter-test coefficients of variation of 8% and 10% for PTFE and PEEK respectively.

The sensitivity of the test presented herein creates some opportunities and highlights some areas for potential further advancement. Testing demonstrated the ability to detect differences in measured peel force due to tape bending. Further study may be able to quantify the force required to bend the tape during testing which would enable higher precision measurement of the true peel force being generated. A target application of the presented method is microfabricated devices, which are inherently small, narrower than the tape. Testing of films that are a fraction of the width of the tape will also provide confirmation that this technique is applicable to small-scale devices. Controlled variation of the peel velocity could potentially be employed to increase the peel force in a single test, allowing one test to measure the continuous relation between peel force and peel rate. Additionally, increasing the peel rate through one test could initiate peeling between different layers allowing the identification of the failure load.

## Data Availability

The data produced by this work are available from first author on reasonable request.
